# “Adding new Molecular Insights to a given Endophenotype: the Relevance of Epigenetics in Environmental Stress Response”

**DOI:** 10.1192/j.eurpsy.2022.100

**Published:** 2022-09-01

**Authors:** F. Rusconi, E. Romito, E. Toffolo, C. Forastieri, E. Battaglioli

**Affiliations:** 1 University of Milan, Dep. Medical Biotechnology And Translational Medicine, SEGRATE, Italy; 2 University of Milan, Dep Medical Biotechnology And Translational Med., Segrate, Italy

**Keywords:** Trauma, epigenetics, Lysine Specific Demethylase 1, Hippocampus

## Abstract

Molecular psychiatry research needs a deeper characterization of emotional and cognitive neural underpinnings, along with a broader recognition of trauma-related circuitries and their involvement in shared pathological endophenotypes. One such endophenotype is unbalanced approach avoidance conflict (AAC), a highly recurrent trait of psychopathology. A translationally validated rodent model of AAC is the elevated plus maze (EPM) test, recently shown to be pharmacologically controlled in human and rodents via homologous neural substrates. Thanks to this test, we identified the involvement of the epigenetic enzyme LSD1 as a molecular restrainer of anxiety. We identified LSD1 aberrant regulation within the hippocampus of suicidal victims, suggesting its broad functional involvement in maladaptive behaviors. Interestingly, thanks to the parallel employment of rodent models, we evaluated a stress-related LSD1 homeostatic regulation that transiently limits memory formation-instrumental gene expression in the hippocampus upon trauma. Our work shed new light on epigenetic processes devoted to trauma resiliency through a negative regulation of anxiety plasticity.

**Disclosure:**

No significant relationships.

